# Repressive and vigilant coping styles in stress and relaxation: evidence for physiological and subjective differences at baseline, but not for differential stress or relaxation responses

**DOI:** 10.3389/fpsyg.2023.1196481

**Published:** 2023-09-01

**Authors:** Anna Exner, Miriam Kampa, Johannes B. Finke, Tobias Stalder, Holger Klapperich, Marc Hassenzahl, Kristian Kleinke, Tim Klucken

**Affiliations:** ^1^Department of Clinical Psychology and Psychotherapy, University of Siegen, Siegen, Germany; ^2^Bender Institute of Neuroimaging, Justus Liebig University Giessen, Giessen, Germany; ^3^Ubiquitous Design/Experience and Interaction, University of Siegen, Siegen, Germany

**Keywords:** repressors, sensitizers, heart rate variability, blood pressure, MMST, stress, relaxation

## Abstract

**Introduction:**

Previous research suggested differential stress reactivity depending on individuals’ coping style, e.g., as classified by the model of coping modes. Specifically, stronger physiological reactivity and weaker subjective stress ratings were found for repressors than for sensitizers. However, it remains to be investigated (i) whether these findings, which are largely based on social stress induction protocols, also generalize to other stressors, (ii) whether repressors vs. sensitizers also exhibit differential stress recovery following the application of a relaxation method, and (iii) which stress reactivity and recovery patterns are seen for the two remaining coping styles, i.e., fluctuating, and non-defensive copers. The current study thus examines stress reactivity in physiology and subjective ratings to a non-social stressor and the subsequent ability to relax for the four coping groups of repressors, sensitizers, fluctuating, and non-defensive copers.

**Methods:**

A total of 96 healthy participants took part in a stress induction (Mannheim Multicomponent Stress Test) and a subsequent relaxation intervention. Subjective ratings of stress and relaxation, heart rate (HR), heart rate variability (HRV), and blood pressure were assessed during the experiment. HR and blood pressure are markers of the sympathetic stress response that can be regulated by relaxation, while HRV should increase with relaxation. To investigate long-term relaxation effects, subjective ratings were also assessed on the evening of testing.

**Results:**

Despite successful stress induction, no differential responses (baseline to stress, stress to relaxation) were observed between the different coping groups on any of the measures. In contrast, a strong baseline effect was observed that persisted throughout the experiment: In general, fluctuating copers showed lower HR and higher HRV than non-defensive copers, whereas repressors reported lower subjective stress levels and higher levels of relaxation during all study phases. No differences in subjective ratings were observed in the evening of testing.

**Conclusion:**

Contrary to previous research, no differential stress reactivity pattern was observed between coping groups, which could be due to the non-social type of stressor employed in this study. The novel finding of physiological baseline differences between fluctuating and non-defensive individuals is of interest and should be further investigated in other stressor types in future research.

## 1. Introduction

Stress has been linked to poor physical and mental health in numerous studies (for detailed reviews see: Schneiderman et al., 2005; O'Connor et al., 2021). The post-processing of stressful experiences (Finke et al., 2022) and the ability to actively relax from perceived stress is an important factor in reducing these harmful effects, such as depression, anxiety, poor quality of life, or burnout (Khoury et al., 2015; Dharmawardene et al., 2016). For these reasons, the way we deal with stress has a significant influence on our health. People differ in the way they cope with stress and anxiety, which can be observed across different situations (Weinberger et al., 1979; Krohne, 2003).

There are different approaches to classifying coping strategies. A common approach is the formation of four groups on the basis of trait anxiety and social desirability (termed ‘defensiveness’) (Weinberger et al., 1979). These two traits are assumed to determine coping patterns in fearful situations. According to this model, repressors are defined as individuals with low anxiety but high defensiveness, sensitizers as individuals with high anxiety and low defensiveness, defensive-anxious as individuals with high scores on both scales, and low-anxious as individuals with low scores on both scales. Despite the wide use of this approach, some problems still need to be considered. The traditional anxiety and social desirability scales neither refer to fear-eliciting situations nor explicitly assess coping behaviors (Krohne, 2003; Paul et al., 2012).

The model of coping modes (MCM) (Krohne, 1993) seeks to overcome these difficulties. It is based on the assumption that people can be distinguished by their dispositional way of dealing with stressful situations. These individual differences are reflected in vigilance (VIG) and cognitive avoidance (CAV), which have been identified by several models of cognitive coping as the dominant dimensions (Miller, 1980; Krohne, 1993; Derakshan et al., 2007). Cognitive avoidance describes the strategy of shielding the organism from threat-related cues, whereas vigilance describes the strategy of actively focusing on such cues (Krohne, 1993; Weinberger, 1995). Vigilance summarizes a class of coping strategies used to reduce uncertainty triggered by unpredictability in fearful situations. Vigilance is thus used by individuals who exhibit an intolerance for uncertainty. Thus, they try to anticipate the course of a negative situation by closely observing and analyzing all related stimuli in order not to be surprised by a negative outcome (Krohne, 1993; Krohne et al., 2000). Cognitive avoidance, on the other hand, summarizes a class of coping strategies aimed at shielding the organism from stressful stimuli that evoke emotional arousal. Cognitive avoidance is thus used by individuals who exhibit an intolerance for emotional arousal and serves as strategy for the reduction of emotional arousal (Krohne, 1993; Krohne et al., 2000). The MCM conceptualizes CAV and VIG as two independent dimensions with the opportunity of classification into repressors (high CAV, low VIG), sensitizers (low CAV, high VIG), fluctuating copers (high on both dimensions), and non-defensive copers (low on both dimensions).

When screening research results for the individual coping groups, the following finding arises. Classifications of repressors and sensitizers based on the MCM and based on the approach by Weinberger et al. (1979), correspond to each other (Krohne et al., 2000). For categorizing fluctuating and non-defensive individuals, however, there is no significant overlap between the two classification systems (Krohne et al., 2000), allowing only studies in which the MCM was used to be considered. For repressors and sensitizers, the body of research is large; in the majority of studies there is a dissociation effect between peripheral physiological responses to stress and subjective ratings of perceived stress between repressors and sensitizers. Repressors tend to show an increased physiological stress response (e.g., heart rate, blood pressure), but lower subjectively perceived stress compared to sensitizers (Newton and Contrada, 1992; Kohlmann et al., 1996; Derakshan and Eysenck, 1997; Rohrmann et al., 2002; Schwerdtfeger and Kohlmann, 2004; Derakshan et al., 2007; Myers, 2010; Paul et al., 2012). A small number of studies, however, has not found this dissociation but instead revealed lower subjective and physiological stress reactivity in repressors (Jørgensen and Zachariae, 2006; Oskis et al., 2019).

Compared to repressors and sensitizers, fluctuating and non-defensive copers have rarely been studied in terms of their stress response. This inhomogeneity is caused by different conceptualizations of coping styles. Most studies rely on the indirect classification approach based on two separate trait questionnaires for anxiety and social desirability (Weinberger et al., 1979). Encouragingly, when group assignment was considered, convergent assignment of repressors and sensitizers could be found, but there was no overlap for the remaining two groups (Krohne et al., 2000). For this reason, only theoretical assumptions can be taken into account: Theoretically, it is assumed that fluctuating individuals show an intolerance for uncertainty as well as for emotional arousal, which leads them to try to protect themselves against both aspects simultaneously. Since this balance cannot be achieved – e.g., simultaneously seeking and avoiding fearful stimuli – it is assumed that they tend to exhibit fluctuating, unstable coping behavior without achieving the desired regulatory effect (Krohne, 1993; Hock and Krohne, 2004). Non-defensive individuals, on the other hand, are understood as individuals who exhibit situation-specific coping behaviors. They are assumed to have neither an increased intolerance for uncertainty nor for emotional arousal. Thus, these individuals could meaningfully adapt their coping behavior to the demands of a given situation, for example, by applying cognitive avoidance in uncontrollable situations and vigilance in situations where controllability can be increased by collecting and monitoring certain anxiety-related information and stimuli (Krohne, 1993; Hock and Krohne, 2004).

Most experimental studies have focused on social stressors, like public speaking, raising the question of whether the results are generalizable to other types of stressors, especially non-social stressors. Indirect information can be derived from data on differential reactivity to medical interventions, which tend to be predominantly physically and less socially threatening. In general, sensitizers indicate more subjective anxiety before invasive medical interventions than patients with other coping dispositions [elective facial surgery (Krohne et al., 1989; Slangen et al., 1993); endoscopic examination (Schwenkmezger et al., 1996)], whereas results on differences in physiological stress parameters are inconsistent. Krohne et al. (1989) observed that non-defensive individuals showed higher physiological stress levels (concentration of free fatty acids in blood) than all other coping groups. Slangen et al. (1993) also found that repressors and sensitizers showed a relatively low stress response (cortisol level) compared to non-defensive individuals and fluctuating copers, with the latter group exhibiting the highest stress response. Schwenkmezger et al. (1996) reported elevated levels of growth hormones in high-vigilant vs. low-vigilant patients, whereas cardiovascular parameters showed no differences between coping groups. The inconsistencies in studies of medical interventions as physiological stressors might be explained by very limited experimental control, e.g., considerable between and within-study differences in the severity of surgical procedures and individual differences in patients’ previous experiences with medical situations. Thus, experimental studies are needed to integrate these results and to better understand the mechanisms underlying stress responses towards non-social stressors.

To our knowledge, there are no experimental studies that focused on the effects of avoidant and vigilant coping on the ability to relax after a stressful event. This asymmetry is surprising, as the post-stress processing and relaxation capacity can contribute significantly to the reduction of negative stress consequences (Khoury et al., 2015; Dharmawardene et al., 2016). Some transfer may be possible from research on the relationship between cognitive load and coping; there is some evidence that sensitizers tend to forget less fear-related information under low cognitive load than other coping style groups (Peters et al., 2012). There is empirical evidence that relaxation can reduce internal sources of cognitive load (for a discussion see Burgess et al., 2017). Thus, it is reasonable to assume that sensitizers might experience less stress reduction from relaxation than the other groups. These findings are partly confirmed by research results on the use of relaxation techniques before medical interventions: Patients who have a tendency towards cognitive avoidance experience the greatest reduction in their stress levels through active relaxation (Lerman et al., 1990; Gattuso et al., 1992; Krohne and El-Giamal, 2008).

The aim of the present study was to investigate group differences between repressors and sensitizers in terms of their subjectively perceived and physiological stress responses. In contrast to previous research, we included the groups of fluctuating and non-defensive individuals according to the MCM categorization scheme to investigate whether potential differences are unique to repressors and sensitizers. Heart rate and blood pressure have been chosen as physiological markers of the sympathetic stress response that can be regulated by relaxation, while heart rate variability should increase with relaxation (Van Diest et al., 2014; Blum et al., 2020). In contrast to previous research that used largely social stressors we applied a stress task without a direct social evaluation, but multiple types of stressors including physically aversive stimuli and focused on the participant’s subsequent ability to relax. In addition, as a follow-up, stress and relaxation ratings were assessed in the evening of testing to test the persistence of relaxation effects. Consistent with the empirical findings and theoretical background, we hypothesized that sensitizers show the greatest self-reported stress, while repressors experience the greatest physiological stress compared to all other groups. Regarding relaxation, we expected that sensitizers and fluctuating individuals show a smaller reduction in physiological and subjectively perceived stress than repressors and non-defensive individuals.

## 2. Materials and methods

### 2.1. Sample

Healthy undergraduates (*N* = 102) were recruited via internal undergraduate mailing lists and online notice boards. Participants received either course credit (*n* = 98) or monetary compensation (15.00€; n = 4) for participation. Two participants dropped out after stress induction, and four participants were excluded because of missing information in the coping questionnaire. Thus, 96 participants (83 women; age: M = 21.42 years, SD = 2.89) were included in the present study. The study was approved by the local ethics committee of the University of Siegen and conducted in accordance with the Declaration of Helsinki. All participants provided written informed consent.

### 2.2. Study design

#### 2.2.1. Procedure

Prior to the laboratory testing, participants completed two online surveys, which included the assessment of trait questionnaires. The actual experiment was conducted in the laboratories of the Department of Clinical Psychology and Psychotherapy at the University of Siegen and consisted of a 5-min baseline measurement, a 5.5-min stress induction, a 15-min relaxation phase, and a 5-min recovery measurement, the latter of which was not included in the present analyses. Physiological data were recorded continuously throughout the test session (see below). Subjective ratings of stress and relaxation were collected at baseline (before the start of stress induction), during stress (in the middle of stress induction), and post relaxation as well as in the evening of testing. A detailed description of all phases will be given in the following sections. For additional information on the original study, see Kampa et al. (2022).

#### 2.2.2. Stress induction

Stress was induced using the Mannheim Multicomponent Stress Test (MMST; Kolotylova et al., 2010), a computer-based stress test that has already shown reliable stress induction on a subjective and physiological level (Reinhardt et al., 2012). The stress test includes presenting aversive sounds (60 randomly timed explosion sounds and noise from 75 to 93 dB) as an acoustic stressor and aversive pictures with fear-eliciting and disgusting scenes as an emotional stressor. Simultaneously, in the foreground, participants performed the PASAT-C (Lejuz et al., 2003), a mental arithmetic task under time pressure, as a cognitive stressor. During the PASAT-C numbers (0–20) were briefly presented (250 ms) with a short duration in between (first half: 3 s, second half: 2 s) and participants were asked to calculate the sum of every two numbers and to enter their results via a numerical mouse keypad, while feedback on correct answers was continuously given. As an additional motivational stressor, participants are told that their reimbursement is reduced for each incorrect response. Since the arithmetic task is performed while being alone in the laboratory and feedback on correct and incorrect answers is given purely computer-aided, the MMST only entails a minor social-evaluative component of stress induction.

#### 2.2.3. Relaxation groups

Participants were randomly assigned to one of two relaxation interventions. The first group perceived a guided virtual reality (VR) intervention, while the second group performed a self-selected relaxation method in a sitting position (e.g., listening to music, reading, breathing exercises etc.). The VR relaxation included a landscape displayed via the HTC Vive pro HMD (HTC Corporation, ViveTM), which was shown as a night scene at the beginning of the intervention and turning into daylight until the end of the intervention (see the Supplementary material for pictures of the relaxation setup and for an impression on the VR scene). Participants were able to look at different parts of the scene by moving their head. The breathing cycle was accompanied by a blue sphere in the VR environment that increased in size for inhalation and decreased in size for exhalation. Additionally, a pillow in the participant’s lap vibrated in sync with the changing size of the blue sphere. A Nexus-10 MKII device (Mind Media, Herten, Netherlands) was used to monitor breathing via a breathing belt around the participant’s chest. The relaxation consisted of different phases. Via headphones participants listened to soothing music, nature sounds and occasionally, a narrator gave instructions on relaxation and respiration. The relaxation started with a familiarization phase including an introduction of the narrator (3 min). Afterwards the sphere reflected a deaccelerated respiratory cycle adapted to each participant by increasing for inhalation and shrinking for exhalation (3 min). In the last phase participants were asked by the narrator to hold their breath after each inhalation and exhale long and deeply afterwards (9 min).

### 2.3. Coping style

The German version of the Mainz Coping Inventory (MCI) (Krohne et al., 2000) was used to assess habitual coping strategies. The MCI measures cognitive avoidance (CAV) and vigilance (VIG) as two separate dimensions of coping with eight threatening situations of varying controllability (four ego-threatening situations: e.g., public speaking; four physically threatening situations: e.g., dentist appointment). For each situation, the MCI contains five items on cognitive avoidance behavior and five items on vigilance behavior in a dichotomous response format (avoidant strategy: e.g. ‘I put on my headphones and listen to music’; vigilant strategy: e.g., ‘I carefully review the topics I’m going to present’). Because we used a non-social stressor with physically threatening components, habitual CAV and VIG scores were calculated as summed scores across all physically threatening situations, as recommended by the authors (Krohne, 1993). Internal consistency was 0.82 for VIG and 0.74 for CAV (Cronbach’s alpha), and there was a negative correlation between the two dimensions (*r* = −0.64, *p* < 0.001), as previously reported (Schwerdtfeger and Rathner, 2016). Eight missing item values were included as indicated in the manual. Sum scores were used to classify participants into the four coping style categories (repressors, sensitizers, non-defensive, and fluctuating copers) by dichotomizing the two dimensions of CAV and VIG by median splits (‘low’: percentile rank below 50% in the current sample; ‘high’: percentile rank above 50% in the current sample) (Table 1).

**Table 1:** Descriptive statistics of the four coping style groups within the sample.

Coping style	*N*	Women	Age	Cognitive avoidance	Vigilance
Repressors	36	26	21.72 (3.32)	15.83 (1.38)	6.22 (2.27)
Sensitizers	36	35	21.31 (2.73)	9.58 (2.60)	13.82 (2.74)
Non-defensive	14	13	21.36 (2.59)	10.00 (1.80)	7.21 (1.89)
Fluctuating	10	9	21.42 (2.89)	14.70 (1.34)	12.0 (2.45)

### 2.4. Subjective ratings

Participants rated their subjective stress and relaxation levels using a Likert scale ranging from 0 (“not stressed,” “not relaxed”) to 9 (“very stressed,” “very relaxed”). For the analyses of subjective stress ratings, two participants had to be excluded due to missing data.

### 2.5. Physiological recordings and processing

Physiological data were recorded with the Biopac MP160 recording system (BIOPAC Systems Inc., Goleta, California, United States) at a sampling rate of 2000 Hz, using a 100C ECG amplifier and a Bionomadix wireless transmitter. Three pre-gelled Ag/Ag-Cl ECG electrodes were placed in lead configuration (ECG II) for electrocardiogram recording. Blood pressure was measured with the Dinamap Pro 300 (General Electric Deutschland Holding GmbH, Frankfurt am Main, Germany) via a blood pressure cuff on the participant’s non-dominant upper arm. Blood pressure measurement was triggered automatically to ensure correct timing. All data were visually inspected and analyzed in Matlab 2020b (The Mathworks Inc., Natick, Massachusetts, United States).

ECG data were analyzed using the Pan-Tompkins algorithm for QRS detection (Pan and Tompkins, 1985) [Matlab implementation (Sedghamiz, 2014)]. Outliers in the intervals between beats (mean+/− 3 standard deviations) were excluded from the analyses. Heart rate was averaged over the entire time span of each phase (baseline, stress, relaxation). Heart rate variability was estimated by applying the root mean square of successive differences (RMSSD) (Shaffer and Ginsberg, 2017) to the inter-beat-intervals. To ensure high data quality, 19 participants had to be excluded from the analysis of cardiac activity because of highly noisy data or serious artefacts, leaving n = 77 participants for the analysis of HR and RMSSD (repressors: 30, sensitizers: 29, non-defensive: 10, fluctuating: 8). Two participants had to be excluded from the analysis of blood pressure because of missing recordings due to technical problems, leaving n = 94 participants for analysis.

### 2.6. Statistical analyses

To examine responses to stress induction and relaxation, repeated-measures analyses of variance (ANOVA) were conducted for the subjective and physiological measures in a design of coping style (repressors vs. sensitizers vs. non-defensive vs. fluctuating) × time (baseline vs. stress vs. relaxation). We controlled for effects between relaxation groups by additionally including this factor in the analysis.^1^ For post-processing in the evening, univariate ANOVAs were conducted for the stress and relaxation ratings to compare the four coping styles. Additional post-hoc tests were conducted to evaluate group differences. All analyses were conducted in SPSS 28 (SPSS 28.0 for Windows, SPSS Inc., Chicago, IL) with an alpha (α)-level of 0.05.

## 3. Results

### 3.1. Manipulation check

**Figure 1:**
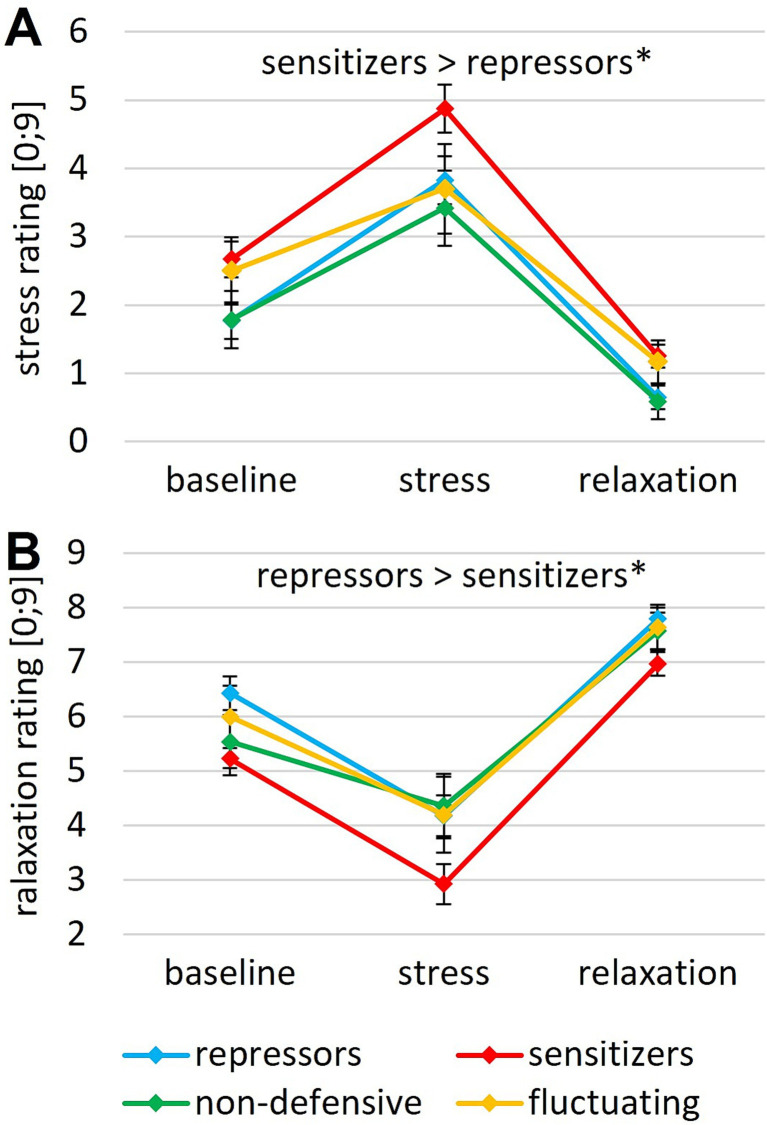
Subjective rating of perceived **(A)** stress and **(B)** relaxation after baseline, during stress induction and after relaxation for different coping styles. Error bars indicate the SEM; ** *p* < 0.01, * *p* < 0.05.

**Figure 2:**
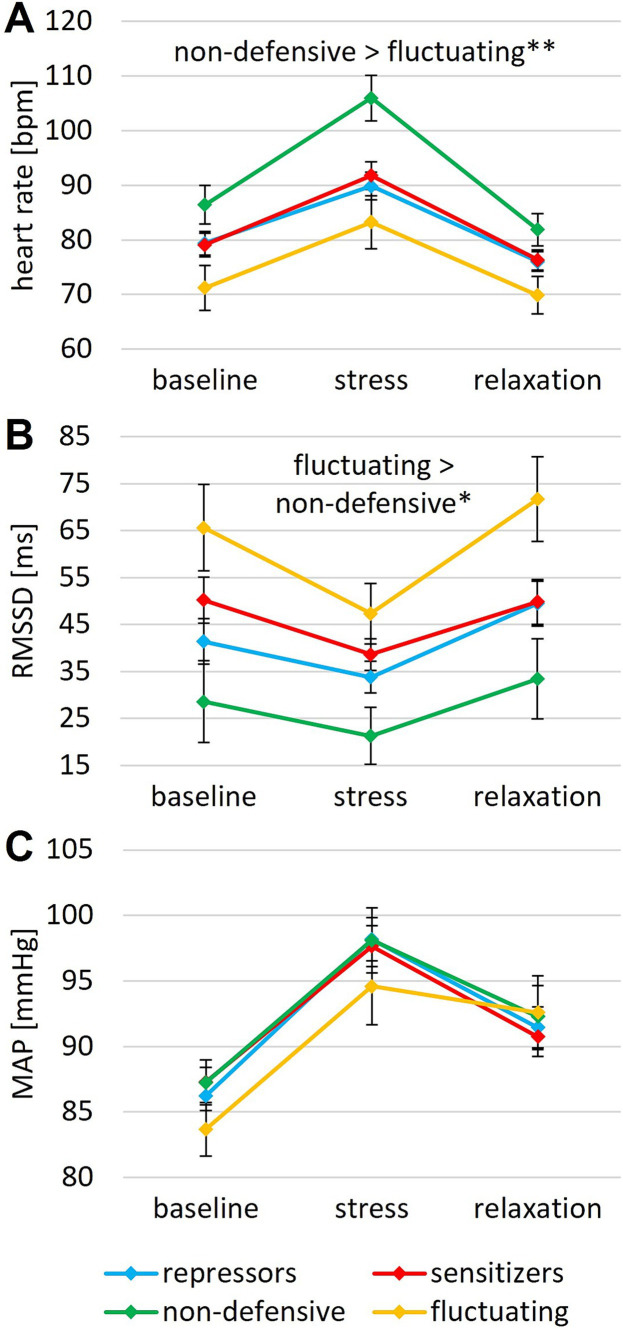
**(A)** Heart rate, **(B)** RMSSD, and **(C)** mean arterial blood pressure during baseline, stress and relaxation for different coping styles. Error bars indicate the SEM; **p < 0.01, *p < 0.05.

All ANOVAs revealed a significant main effect of time (Greenhouse–Geisser corrected; HR: *F* (1.38, 101.97) = 131.23, *p* < 0.001, *η*_p_^2^ = 0.639; RMSSD: *F* (1.75, 125.65) = 33.45, *p* < 0.001, η_p_^2^ = 0.317; blood pressure: *F* (1.72, 152.87) = 64.69, *p* < 0.001, η_p_^2^ = 0.580; stress rating: *F* (1.69, 150.48) = 122.92, *p* < 0.001, *η*_p_^2^ = 0.580; relaxation rating: *F* (1.82, 165.52) = 134.91, *p* < 0.001, *η*_p_^2^ = 0.597) (Figures 1, 2). Bonferroni-adjusted post-hoc analyses revealed significantly higher heart rate, lower heart rate variability, higher blood pressure, higher subjective ratings of stress and lower ratings of relaxation for the stress induction phase than for baseline (HR: M_diff_ = 13.68, *p* < 0.001; RMSSD: M_diff_ = −11.21, *p* < 0.001; blood pressure: M_diff_ = 11.02, *p* < 0.001; stress rating: M_diff_ = 1.77, *p* < 0.001; relaxation rating: M_diff_ = −1.88, *p* < 0.001). In addition, reverse effects were found between the stress and relaxation phases (HR: M_diff_ = −16.68, *p* < 0.001; RMSSD: M_diff_ = 15.85, *p* < 0.001; blood pressure: M_diff_ = −5.37, *p* < 0.001; stress rating: M_diff_ = −3.04, *p* < 0.001; relaxation rating: M_diff_ = 3.58, *p* < 0.001). These effects demonstrate successful stress induction and successful relaxation across the sample.

### 3.2. Subjective ratings

Significant main effects of coping style were revealed for both stress (*F* (3, 89) = 3.26, *p* = 0.025, *η*_p_^2^ = 0.099) and relaxation ratings (*F* (3, 91) = 3.44, *p* = 0.020, *η*_p_^2^ = 0.102) (Figure 1). No interactive effects reached significance (all *p* > 0.429). A Bonferroni-adjusted post-hoc analysis revealed significantly higher stress and lower relaxation scores for sensitizers than for repressors (stress rating: M_diff_ = 1.00, *p* = 0.049; relaxation rating: M_diff_ = −1.09, *p* = 0.016). Univariate ANOVAs for stress and relaxation ratings on the evening of testing revealed no significant group differences (all *p*s > 0.230).

### 3.3. Physiological measures

For HR, a significant main effect of coping style (*F* (3, 74) = 3.89, *p* = 0.012, *η*_p_^2^ = 0.136) and a significant time × coping style interaction (Greenhouse–Geisser corrected; *F* (4.13, 101.97) = 2.60, *p* = 0.039, *η*_p_^2^ = 0.095) were found (Figure 2A). Bonferroni-adjusted post-hoc analysis revealed significantly higher HR in non-defensive than in fluctuating copers (M_diff_ = 16.34, *p* = 0.008). As post-hoc tests for the significant interaction, univariate ANOVAs were conducted for stress response (HR_stress_ – HR_baseline_) and relaxation response (HR_relax_ – HR_stress_) with coping styles as the between-subject factor. No statistically significant effects were found (stress: *F* (3, 45) = 1.65, *p* = 0.184; relaxation: *F* (3, 45) = 1.60, *p* = 0.196).

Regarding HRV, the ANOVA revealed a significant main effect for coping style (*F* (3, 72) = 3.57, *p* = 0.018, *η*_p_^2^ = 0.129) (Figure 2B). Bonferroni-adjusted post-hoc analysis revealed a significantly higher RMSSD for fluctuating than for non-defensive copers (M_diff_ = 33.77, *p* = 0.014). Additionally, a significant time × relaxation group interaction (*F* (1.75, 125.64) = 3.76, *p* = 0.031, *η*_p_^2^ = 0.050) was found. Post-hoc analyses showed a significantly higher HRV during relaxation for the guided intervention than for the self-selected intervention (*F* (1, 72) = 4.30, *p* = 0.042, *η*_p_^2^ = 0.056), which was expected due to the guidance toward slow, regular breathing in the guided intervention.

In the ANOVA on mean arterial blood pressure, no other effects (apart from the main effect for time) reached significance (Figure 2C).

### 3.4. Additional analyses

Since one could argue that the classification of individuals close to the median is highly dependent on the sample, in addition to the above analyses, further analyses were conducted following a classification of the four groups based on quartile splits of the two dimensions of CAV and VIG (“low”: percentile rank below 25% in the current sample; “high”: percentile rank above 75% in the current sample) to confirm potential findings. This procedure reduced the group sizes to 18 repressors, 16 sensitizers, 3 non-defensive, and 1 fluctuating subjects. Because of the reduced size, only results between repressor and sensitizer were calculated. The results essentially confirmed the previous results between repressors and sensitizers: (1) No significant difference was found in stress or relaxation responses between repressors and sensitizers for physiological or subjectively perceived stress and (2) Sensitizers showed a higher subjective stress level throughout the entire experiment as compared to repressors (stress rating: *F* (1, 30) = 5.21, *p* = 0.030, *η*_p_^2^ = 0.148).

## 4. Discussion

This study investigated stress reactivity to non-social stress, as well as the post-stress relaxation ability and post-processing after experiencing stress of individuals with different coping styles. Overall, results replicate the typically lower subjective stress ratings for repressors than for sensitizers. Contrary to expectation, there were no significant differences between repressors and sensitizers in the physiological stress responses. However, there was a significant difference in physiological measures between non-defensive and fluctuating copers at baseline, during stress induction and during relaxation, with non-defensive individuals overall showing a higher HR and lower HRV than fluctuating individuals. No group differences were observed in the magnitude of stress reduction neither through relaxation nor at post-processing.

### 4.1. Sensitizers experienced more subjective stress

Repressors reported lower stress levels than sensitizers, which is in line with data showing that sensitizers report higher subjective stress before and during experimental stress inductions (Newton and Contrada, 1992; Kohlmann et al., 1996; Derakshan and Eysenck, 1997; Rohrmann et al., 2002; Schwerdtfeger and Kohlmann, 2004; Derakshan et al., 2007; Myers, 2010; Paul et al., 2012; Oskis et al., 2019). Additional evidence can be found in research on the stress of having to undergo a medical intervention wherein sensitizers show an increased stress experience at several measurement time points before the intervention (Krohne et al., 1989; Slangen et al., 1993; Schwenkmezger et al., 1996). The stress response as defined by the change in subjective stress rating on the other hand did not differ between coping groups, which has also been reported previously (Jørgensen and Zachariae, 2006; Oskis et al., 2019).

### 4.2. Repressors showed no increased physiological stress responses

The majority of studies reported a dissociation between subjective ratings and physiological stress responses between repressors and sensitizers (Schwerdtfeger and Kohlmann, 2004; Myers, 2010). A dissociation was found here in the form of a lower subjective stress rating but an equally strong physiological stress response. In contrast, the physiological stress response, i.e., baseline-to-stress changes in HR, HRV and mean arterial blood pressure, did not show significant differences between repressors and sensitizers. Even though the majority of research findings point to significant differences in stress reactivity (Schwerdtfeger and Kohlmann, 2004; Myers, 2010), the same pattern has been observed by other studies before (Oskis et al., 2019). Importantly, this previous research exclusively used social stressors, such as a speech in front of an audience, which thus mainly presented threat to self-esteem. Non-socially threatening situations, i.e., also including a physically aversive element, can be assumed to be inherently less controllable than socially threatening situations (Schmukle et al., 2000) and might thus be associated with divergent stress reactivity patterns.

### 4.3. Relaxation responses did not differ between coping groups

With respect to the relaxation response (i.e., the change between experienced stress during stress induction and after relaxation), no differences between coping groups were observed in this study. Consistently, no coping group differences were observed for subjective stress ratings in the evening of testing. Thus, contrary to the evidence from relaxation interventions prior to medical procedures (Lerman et al., 1990; Gattuso et al., 1992; Krohne and El-Giamal, 2008), sensitizers were shown to benefit no less from relaxation than repressors. An important difference to these studies, however, is that the relaxation techniques were applied before the medical intervention, thus they must be assumed to mainly influence baseline levels and/or the cognitive appraisal of upcoming stressors. By contrast, the focus in the present study was on inducing relaxation after a stressful experience. Here, there were no differences between the coping groups. Because coping groups often differed in their response to social stress in previous work, it would be interesting for future studies to examine the ability to relax following a social stress induction.

### 4.4. Fluctuating and non-defensive individuals differ in their baseline physiological stress level

Concerning the two groups of fluctuating and non-defensive individuals, we found a significant difference in physiological measures at baseline and throughout the entire experiment. Fluctuating individuals showed an increased heart rate variability and a lower HR compared to non-defensive copers. For these findings, we find supporting evidence in research on medical interventions. Krohne et al. (1989) observed that non-defensive individuals showed higher physiological stress levels (concentration of free fatty acids in blood) than all other coping groups. Slangen et al. (1993) also found that repressors and sensitizers showed a relatively low stress response (cortisol level), whereas non-defensive individuals demonstrated a significantly elevated stress response and fluctuating individuals showed the highest stress response. Relating these results to the MCM, the assumed intolerance of uncertainty and arousal as basis for the dimensions of vigilance and cognitive avoidance might lead to a differential adoption of coping resources in fluctuating vs. non-defensive copers: Since non-defensive individuals, with low scores on both the vigilance and cognitive avoidance dimensions are able to tolerate both arousal and uncertainty, they might have had to adopt few coping strategies over the course of their lives, while individuals who score high on both dimensions – the fluctuating – had to learn a huge variety of coping strategies, which could be used adaptively. Support for this theory can be found in a latent class analysis by Schmukle et al. (2000), wherein the fluctuating individuals are identified as individuals who adapt their coping strategy depending on the situation. In conclusion, the group of fluctuating individuals might consist of individuals with a variety of coping strategies, which might be beneficial for non-socially experienced stress. Further research on the coping styles of fluctuating and non-defensive individuals is clearly needed to understand how these coping styles are used in different situations and to draw conclusions about the extent to which one style may be more adaptive than the other.

Emotion regulation and coping processes are thought to be related to a person’s ability to adjust HRV in the short-term depending on the circumstances (Thayer and Lane, 2000). Higher HRV at rest has usually been associated with a more effective emotion regulation (Thayer and Lane, 2009; Park and Thayer, 2014; Balzarotti et al., 2017). Since dysfunctional emotion regulation is often associated with the development and maintenance of mental health problems (Sheppes et al., 2015) and research on fluctuating and non-defensive copers is sparse, it might be interesting for future research to further investigate these two coping styles in terms of their emotion regulation abilities.

### 4.5. Limitations

The presented study has some limitations that should be mentioned. The group sizes for fluctuating and non-defensive individuals are considerably smaller than for repressors and sensitizers. This underrepresentation has also been found in previous studies (Peters et al., 2012), but results in reduced power of the analyses. However, when looking at the data more closely, one can assume that the main expected effects – i.e. the differences between repressors and sensitizers in the stress response – would presumably not be found even with a larger sample. The standardized effect size in the stress rating is 0.2, which is a very small effect. In contrast, in the physiological response, exemplified by heart rate, a moderate effect of about 0.4 was found, but pointing in the opposite direction rather than the expected one. Further research is needed to determine whether the cause of the direction of this effect is due to the nature of the stress induction or to selection effects of the sample. Additionally, sexes are unequally distributed across coping groups. The high number of male participants in the repressors group might cause a bias in the results. Future studies might want to control for such biases by aiming for a recruitment of similar distributions between coping styles. Another limitation is that the differences found between the groups of fluctuating and non-defensive individuals rely on the classification by Krohne (1993). Studies based on the classification by Weinberger et al. (1979) will most likely come to different results and conclusions since group assignment hardly overlaps. It might be interesting for future research to additionally examine stress and relaxation responses using this classification to determine if there are differences according to this classification. Another limitation is that our study sample consisted of a healthy student cohort with an associated lower variance in coping styles than might be found in a clinical population. Furthermore, when interpreting HRV, it is important to keep in mind that HR and HRV are inversely correlated (Kazmi et al., 2016), making it difficult to interpret group differences in HRV independently of HR. Correspondingly, an additional ANCOVA controlling for baseline differences in HR did not reveal a significant group difference in HRV. This finding further highlights the importance of carefully evaluating the specificity of HRV effects in future research. Another limitation relates to the generalizability of the study: future studies should investigate whether the described effects can also be observed in populations other than a healthy student sample and for other non-social stress inductions. Overall, the question arises whether there might be differential effects for the individual coping styles depending on the type of stressor. This could be addressed in future studies by looking at different stressors individually (e.g., acoustic stressors, aversive images, emotional stressors) and by also systematically varying the controllability of the stressors.

### 4.6. Conclusion

In summary, we found support for the dissociation between subjective ratings and physiology in repressors in the form of a lowered subjective perception of stress throughout the entire experiment. Additionally, no substantial differences in stress reactivity were observed between coping groups, neither on a physiological nor on a subjective level. As the present study involved a non-social stressor, this might be viewed as tentative indication for the notion that these inter-individual differences only arise when stress induction involves pronounced social threat. However, additional corroboration by future research is needed here. The differences at baseline that persist throughout the experiment could be interesting for future research and shows the importance of examining all four coping groups.

## Data availability statement

The raw data supporting the conclusions of this article will be made available by the authors, without undue reservation.

## Ethics statement

The studies involving humans were approved by local ethics committee of the University of Siegen. The studies were conducted in accordance with the local legislation and institutional requirements. The participants provided their written informed consent to participate in this study.

## Author contributions

AE: conceptualization, methodology, formal analysis, visualization, and writing – original draft. MK: conceptualization, methodology, software, formal analysis, and writing – review and editing. JF and TS: conceptualization, software, investigation, and writing – review and editing. HK: conceptualization, software, and writing – review and editing. MH and TK: conceptualization, supervision, funding acquisition, and writing – review and editing. KK: formal analysis and writing – review and editing. All authors contributed to the article and approved the submitted version.

## Funding

This work was funded by the Bundesministerium für Bildung und Forschung (BMBF, Funding number: 16SV8068) and a scholarship for doctoral students from the Faculty of Education, Architecture, and the Arts of the University of Siegen.

## Conflict of interest

The authors declare that the research was conducted in the absence of any commercial or financial relationships that could be construed as a potential conflict of interest.

## Publisher’s note

All claims expressed in this article are solely those of the authors and do not necessarily represent those of their affiliated organizations, or those of the publisher, the editors and the reviewers. Any product that may be evaluated in this article, or claim that may be made by its manufacturer, is not guaranteed or endorsed by the publisher.

## Footnotes

^1^To ensure that observed effects were not confounded by a random imbalance in the distribution of coping styles across the relaxation groups, a chi-square test was conducted. There was no statistically significant correlation between relaxation group and coping style, χ^2^(3) = 4.02, *p* = 0.259, φ = 0.21.
