# Supplementary material for: Repressive and vigilant coping styles in stress and relaxation: evidence for physiological and subjective differences at baseline, but not for differential stress or relaxation responses
Source: Front Psychol. 2023 Sep 1;14:1196481. doi: 10.3389/fpsyg.2023.1196481 (PMC10502326; doi:10.3389/fpsyg.2023.1196481)
Supplement: Supplementary file 1 [file Data_Sheet_1.pdf]

Supplementary material

Corresponding manuscript:

Exner A, Kampa M, Finke JB, Stalder T, Klapperich H, Hassenzahl M, Kleinke K and Klucken T (2023) Repressive and vigilant coping styles in stress and relaxation: evidence for physiological and subjective differences at baseline, but not for differential stress or relaxation responses. *Front. Psychol*. 14:1196481. doi: 10.3389/fpsyg.2023.1196481

1. Virtual reality (VR) relaxation group

1.1. The setup

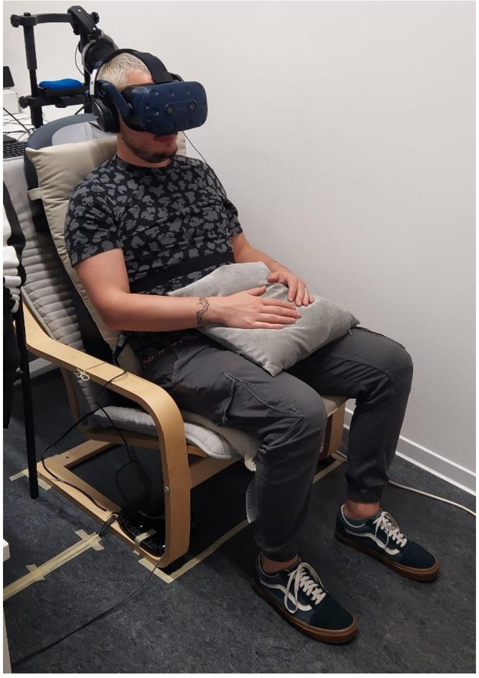

Fig. 1: Relaxation setup in the VR relaxation group

1.2. The VR scene

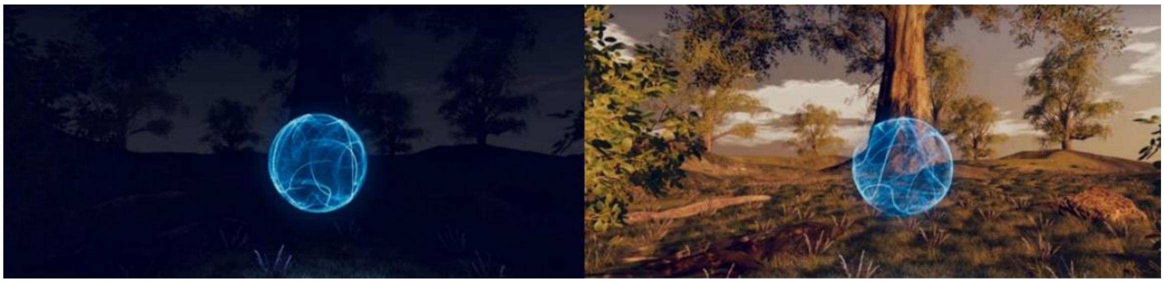

Fig. 2: The natural scene in the VR environment. The scene starts as a night scene (left) turning into daylight (right). The blue sphere gives feedback on the breathing cycle: size increases during inhalation and shrinks during exhalation (Kampa et al., 2022)

1.3. User experiences during the VR relaxation

All participants described that the VR environment helped them to relax. Some especially mentioned the natural environment, which was beneficial for relaxation. The setting was recognized by all as a 'safe space'. Above all, the high immersion of the VR helped to escape from everyday life and to focus on oneself and one's own breath. All participants used the opportunity to set their own focus in the VR environment and, for example, to look at the sky, or even to close their eyes in order to concentrate entirely on the background music and the speaker's instructions.

Reference

Kampa, M., Finke, J., Stalder, T., Bucher, L., Klapperich, H., Mertl, F., Zimmer, C., Geiger, C., Hassenzahl, M., and Klucken, T. (2022). Facilitating relaxation and stress reduction in healthy participants through a virtual reality intervention: study protocol for a non-inferiority randomized controlled trial. *Trials* 23, 380. doi: 10.1186/s13063-022-06307-8.
